# Some Account of an Hysterical Affection of the Vocal Apparatus, with Several Cases

**Published:** 1844-07

**Authors:** Oscar Clayton


*Some Account of an Hysterical Affection of the Vocal Apparatus, with several Cases.* By Oscar Clayton, Esq.—The cases occurred in a charitable institution for female children, from the age of nine to that of fourteen. The cases were seventeen in number. They began with feverish and ended with hysterical characters. The main feature was spasmodic cough, which, in a few weeks, changed to sounds varying in the different patients; in some resembling the double action of a large saw; in two, a shrill screaming expiration, following a quick catching inspiratory effort, much resembling the cry of a peacock; in another, the sound was such as is produced by blowing into a small metallic tube. In fact, it is difficult to conceive the dissonance and constancy of these sounds.

All kinds of remedies failing, the patients were sent home, where they recovered, but on coming together again, the same farce was re-enacted. An attempt was made to frighten the complaint away, by calling the girls together, and informing them that, if not better the next morning, a hot iron would be applied upon their throats. Most of them ran away, but the complaint was not got rid of. A hot spatula on the throat did some good; time did more.—*Med. Chi. Rev.*, Jan.

